# Quantitative PCR and Digital PCR for Detection of* Ascaris lumbricoides* Eggs in Reclaimed Water

**DOI:** 10.1155/2017/7515409

**Published:** 2017-03-09

**Authors:** Lucrecia Acosta Soto, Ana Belén Santísima-Trinidad, Fernando Jorge Bornay-Llinares, Marcos Martín González, José Antonio Pascual Valero, Margarita Ros Muñoz

**Affiliations:** ^1^Department of Soil and Water Conservation and Organic Waste Management, Centro de Edafologia y Biologia Aplicada del Segura (CEBAS-CSIC), Campus de Espinardo, P.O. Box 164, Espinardo, 30100 Murcia, Spain; ^2^Área de Parasitología, Departamento de Agroquímica y Medio Ambiente, Universidad Miguel Hernández de Elche, Ctra Valencia Km 8.7, San Juan, 03550 Alicante, Spain; ^3^Aguas de Murcia, C/Rincón de San Antón, Llano de Brujas, 30161 Murcia, Spain

## Abstract

The reuse of reclaimed water from wastewater depuration is a widespread and necessary practice in many areas around the world and must be accompanied by adequate and continuous quality control.* Ascaris lumbricoides *is one of the soil-transmitted helminths (STH) with risk for humans due to its high infectivity and an important determinant of transmission is the inadequacy of water supplies and sanitation. The World Health Organization (WHO) recommends a limit equal to or lower than one parasitic helminth egg per liter, to reuse reclaimed water for unrestricted irrigation. We present two new protocols of DNA extraction from large volumes of reclaimed water. Quantitative PCR (qPCR) and digital PCR (dPCR) were able to detect low amounts of* A. lumbricoides* eggs. By using the first extraction protocol, which processes 500 mL of reclaimed water, qPCR can detect DNA concentrations as low as one* A. lumbricoides *egg equivalent, while dPCR can detect DNA concentrations as low as five* A. lumbricoides *egg equivalents. By using the second protocol, which processes 10 L of reclaimed water, qPCR was able to detect DNA concentrations equivalent to 20* A. lumbricoides* eggs. This fact indicated the importance of developing new methodologies to detect helminth eggs with higher sensitivity and precision avoiding possible human infection risks.

## 1. Introduction

The reuse of wastewater after the depuration process is a widespread and necessary practice in many areas around the world, especially in areas prone to water scarcity. Reclaimed water can be used in agriculture and become a risk for human health due to the presence of an array of pathogens and pollutants in the reclaimed water used for irrigation [1–3]. In particular, helminth ova are capable of surviving months in water, or even years in soil, and are a potential concern wherever wastewater or biosolids are reused [4].

In 1989, the World Health Organization (WHO) [5] drew attention of health implications due to helminth infections associated with inadequate water quality and sanitation [2, 3]. Anemia, malnutrition, cognitive impairment, and gastrointestinal or pulmonary complaints are some of the problems associated with intestinal helminth infections [2, 6, 7].* Ascaris lumbricoides *is one of the nematode species that cause soil-transmitted helminthic diseases (STH) and, globally, it affects over 819 million people. Of the 4.98 million years lived with disability (YLDs) attributable to STH, roughly 1.10 million YLDs were attributable to* A. lumbricoides* [8]. In addition, deaths from STH are attributable to heavy* A. lumbricoides *infection in children under 10 years of age [9].

Due to their environmental hardiness, the presence of parasitic helminth eggs as an indicator of sanitary risk is one of the water quality parameters recommended by the WHO [10]. An upper limit of one helminth egg per liter is recommended for reclaimed water to be judged suitable for unrestricted use [11, 12]. Following these recommendations, the modified Bailenger method was proposed as a reference method to detect a maximum limit of one intestinal helminth egg per 10 liters of water for diverse reuse in urban, agricultural, industrial, or environmental contexts [13, 14]. This method is considered to be time consuming (minimum 72 hours), not very sensitive, and involves subjective morphological identification and quantification of nematode eggs by optical microscopy after flotation [15].

Molecular techniques such as quantitative PCR (qPCR) or the recently developed digital PCR (dPCR) are faster and more precise techniques for identification of species and could facilitate clinical diagnosis and improve the reliability and objectivity of analytical methods. However, one of the key points for the success of molecular methods is to find the most appropriate method of DNA extraction to obtain a high quality DNA yield, with a minimum amount of PCR inhibitors [16].

Some authors have used qPCR to detect human intestinal parasites in water samples [17] and in feces [18–24], but not from reclaimed water. Nevertheless, non-human helminths parasites have been detected in fresh water [25], soil, and wastewater samples by qPCR [26].

Digital PCR (dPCR) has been used primarily in clinical diagnostics [27–29] and is a promising step forward, as this technology provides absolute quantification of DNA without the need for a standard curve [30, 31]. Absolute quantification via dPCR is achieved by partitioning the sample into a large number of individual reactions, then assessing the proportion of positive reactions [32]. Also, dPCR improves sensitivity when quantifying low concentrations of target genes in highly concentrated background DNA samples [27]. There have been no published methods for detecting* A. lumbricoides *ova in reclaimed water by qPCR or dPCR.

The success of PCR detection methods hinges on the techniques used to extract DNA. Optimal sensitivity is attained when extraction techniques are able to recover a large amount of target DNA from the sample media without also extracting PCR inhibitors [16].

The aim of this study is to demonstrate if two molecular techniques (qPCR and dPCR) can be used to detect* A. lumbricoides *eggs in reclaimed water. For this purpose, two new protocols of DNA extraction were developed to obtain sufficient quality to detect* A. lumbricoides *eggs by qPCR and dPCR.

## 2. Material and Methods

### 2.1. Experimental Design

The experimental design consisted of three steps: (1) sterile bidistilled water (50 *μ*L) was seeded with different amounts of* A. lumbricoides* eggs, and DNA extraction protocol and detection and quantification by qPCR and dPCR were optimized; (2) reclaimed water (500 mL and 10 L) was seeded with different amounts of* A. lumbricoides* eggs, for assaying DNA extraction of* A. lumbricoides* eggs and detection and quantification by qPCR and dPCR; and (3) reclaimed water (10 L) was seeded with different amounts of* A. lumbricoides* eggs and they were externally analyzed to detect helminth eggs in water by the modified method of Bailenger (WHO) by three ENAC (Spanish National Accreditation Body) accredited laboratories (see the following).

*Chart of Extraction and Detection*

*Control*

  Volume: 50 *μ*L of bidistilled water.
  Number of eggs: 1 (×5 replicates); 2 (×5 replicates); 5 (×5 replicates); 10 (×5 replicates); 20 (×5 replicates); and 50 (×5 replicates).
  Extraction: 1 × 50 *μ*L each replicate.
  qPCR dilutions: 1 : 5, 1 : 10, 1 : 20 dilutions of each replicate.
  Template: qPCR (3 × 5 *μ*L aliquots of each dilution); dPCR (4 *μ*L each replicate).
  Final volume: qPCR (25 *μ*L); dPCR (16 *μ*L).

*Protocol  1*

  Volume: 500 mL of reclaimed water.
  Number of eggs: 1 (×5 replicates); 5 (×5 replicates); and 10 (×5 replicates).
  Extraction: 1 × 50 *μ*L each replicate.
  qPCR dilutions: 1 : 5, 1 : 10, 1 : 20 dilutions of each replicate.
  Template: qPCR (3 × 5 *μ*L aliquots of each dilution); dPCR (4 *μ*L each replicate).
  Final volume: qPCR (25 *μ*L); dPCR (16 *μ*L).

*Protocol  2*

  Volume: 10 L of reclaimed water.
  Number of eggs: 1 (×5 replicates); 2 (×5 replicates); 5 (×5 replicates); 10 (×5 replicates); 20 (×5 replicates); and 50 (×5 replicates).
  Extraction: 6 × 50 *μ*L sample of each replicate.
  qPCR dilutions: 1 : 5, 1 : 10, 1 : 20 dilutions of each replicate.
  Template: qPCR (3 × 5 *μ*L aliquots of each dilution); dPCR (4 *μ*L each replicate).
  Final volume: qPCR (25 *μ*L); dPCR (16 *μ*L).

### 2.2. Source of* A. lumbricoides* Eggs

The* A. lumbricoides *eggs were extracted from infected human feces provided by Hydrolab S.L. Human feces were inactivated in 70% ethanol and preserved in saline solution where* A. lumbricoides *eggs were isolated under a magnifying glass and placed into 1.5 mL tubes with nuclease-free bidistilled water (bdW). Aliquots of 1, 5, 10, 20, 50, and 100 eggs were stored at 4°C until use.

### 2.3. DNA Extraction from Bidistilled Water (bdW) Seeded with* A. lumbricoides* Eggs

Five replicates from 1, 5, 10, 20, or 50* A. lumbricoides *eggs with 50 *μ*L of bdW each were separated in 25 tubes. Finally, five batches with six samples each were extracted: five replicates (bdW seeded) and an extra sample without eggs by batch (bdW negative control). Each sample was washed twice with 500 *μ*L of phosphate buffered saline (PBS, Sigma-Aldrich, Poole, Dorset, UK), vortexing for 30 s and centrifuged at 14,000 rpm for 10 min. The supernatants were removed and pellets were resuspended in 50 *μ*L of bdW and three borosilicate glass beads were added. Samples were subjected to three cycles of freezing with liquid nitrogen for 5 s followed by thawing in a 56°C water bath for 5 min and vortexed for 40s and speed setting of 6.0 m s^−1^ using the FAST-PREP® 24 instrument (Mp Biomedicals, Irvine, CA, USA). Samples were centrifuged at 8,000 rpm for 1 min. Later, DNA concentration was measured by Quant-iTPicoGreen dsDNA assay kit (Invitrogen, Carlsbad, CA, USA).

### 2.4. DNA Extraction from Reclaimed Water Seeded with* A. lumbricoides *Eggs

Reclaimed water was provided by Murcia Este Wastewater Treatment Plant (WWTP). The Murcia Este WWTP processes urban wastewater using activated sludge (type A2O) which allows a significant removal of nutrients (N and P) in the treated water. An anaerobic digestion process stabilizes the excess sludge generated. The reclaimed physicochemical water parameters were pH 7.84 ± 0.09, 3.5 ± 0.48 nephelometric turbidity units (NTU), 2.24 ± 0.12 mS cm^−1^ conductivity, 11.42 ± 5.96 mg L^−1^ total suspended solids (SS), 7.39 ± 1.96 mg L^−1^ biochemical oxygen demand (BOD), 36 ± 7.74 mg L^−1^ chemical oxygen demand (COD), 13.71 ± 3.72 mg L^−1^ total nitrogen (TN), and 2.48 ± 0.06 total phosphorous (TP).

#### 2.4.1. Protocol  1 (500 mL of Reclaimed Water)

Three batches of five reclaimed water samples (500 mL) each were seeded with 1, 5, or 10* A. lumbricoides* eggs (five replicates per sample). Reclaimed water not seeded was used as negative control in each batch. To retain the helminth ova, seeded reclaimed water was filtered through a 47 mm diameter Durapore® filter with a light mesh of 0.65 *μ*m (Millipore Corp., Bedford, MA, USA). This filter was mechanically and enzymatically digested by adding 1 g of glass beads (425–600 *μ*m diameter), 920 *μ*L of NET 10 (10 mM NaCl, 10 mM EDTA, and 10 mM Tris HCl), 40 *μ*L of proteinase K (30 mg mL^−1^), and 40 *μ*L of 10% sodium dodecyl sulfate (SDS) (Sigma-Aldrich, Poole, Dorset, UK). Then, samples were subjected to three cycles of freezing with liquid nitrogen for 5 s followed by thawing in a 56°C water bath for 5 min and vortexed at maximum speed in a Vortex Genie 2 machine for 2 min (MoBio Laboratories, Inc., Solana Beach, CA, USA). The samples were centrifuged at 8,000 rpm for 1 min and the supernatant (containing DNA) was transferred to new tubes. DNA was extracted by conventional phenol : chloroform : isoamyl alcohol purification (25 : 24 : 1) and ethanol precipitation and further eluted in 50 *μ*L of bdW.

#### 2.4.2. Protocol  2 (10 L of Reclaimed Water)

Five batches of 10 L of reclaimed water were seeded with 1, 5, 10, 20, and 50* A. lumbricoides* eggs (five replicates per sample). Reclaimed water not seeded was used as control in each batch. This protocol was similar to protocol 1, but with some differences due to the higher amount of water to be filtered.

Ten liters of reclaimed water was filtered through two Durapore filters (5 L per each filter) of 97 mm diameter with 5 *μ*m light mesh (Millipore Corp., Bedford, MA, USA). Each filter was then introduced into a 15 mL tube with 2720 *μ*L of CTAB buffer (2% hexadecyl-trimethyl-ammonium bromide, 1.4 M NaCl, 20 mM EDTA, and 100 mM Tris pH 8.0), 2% polyvinylpolypyrrolidone (PVPP), 40 *μ*L of proteinase K (30 mg mL^−1^), 240 *μ*L of 10% SDS, and 2 g of glass beads (425–600 *μ*m diameter) (Sigma-Aldrich, Poole, Dorset, UK). After this point, each sample was divided into 3 similar aliquots (1 mL each) and the protocol continued as per the 500 mL one (protocol 1), with three cycles of freezing/thawing, centrifugation, DNA purification by phenol : chloroform : isoamyl alcohol and ethanol purification, and the pellet eluted in 50 *μ*L of bdW. For every 10 L of reclaimed water sample, we obtained six 50 *μ*L DNA extracts (2 filters for each 10-liter sample, each filter was divided into 3 samples); and in the case of one of them resulting in qPCR or dPCR amplification, the whole reclaimed water batch was considered to have* A. lumbricoides* present in the sample (positive reaction).

### 2.5. Quantitative PCR (qPCR)

Quantitative PCR (qPCR) amplifications were performed in a 7500 Fast Real-Time PCR system (Applied Biosystems®), using a Microamp® Fast Optical 96-Well Reaction Plate with barcode (Life Technologies, Carlsbad, CA, USA), in a final volume of 25 *μ*L. The final qPCR mixture contained 1x TaqMan Universal Master Mix II (Life Technologies, Carlsbad, CA, USA), 200 *μ*M of each dNTP, 0.3 *μ*M of each primer (Roche Diagnostics, Germany), 0.1 *μ*M of the probe, 0.2 mg mL^−1^ BSA, and 5 *μ*L of DNA sample. The specific primers Alum96F and Alum183R, that amplify an 89 bp fragment of the ITS-1 (located between 18S and 5.8S rRNA genes) sequence with the FAM-labeled probe Alum124T with TMR quencher, were used to detect* A. lumbricoides *[18, 33]. The thermocycling conditions were 95°C for 10 min, followed by 40 cycles of 95°C for 10 s and 60°C for 40 s. To detect inhibition, a TaqMan® Exogenous Internal Positive Control Reagent—containing a preoptimized internal positive control (IPC) with predesigned primers—and a TaqMan probe (Applied Biosystems) were included in all reactions, according to the manufacturer's recommendations. Each run contained one negative (bdW) and one* A. lumbricoides* DNA positive control. Dilutions (1 : 5, 1 : 10, and 1 : 20) of the DNA extracts were used as a template. Each sample was analyzed in triplicate. In order to assess the sensitivity of the assay, the qPCR reactions were considered negative/nondetects if the Ct value exceeded 37. To calculate the copies per microliter, the dilution factor in each case was taken into account.

A standard curve of* A. lumbricoides *DNA was done by cloning the amplified fragment 89 bp fragment of the ITS-1 by specific primers Alum96F and Alum183R [18, 33] with TA Cloning® Kit Dual Promoter (pCR™ II vector) (Invitrogen, Carlsbad, CA) and the plasmid vector obtained was used to transform* Escherichia coli* DH5*α* cells (Invitrogen, Carlsbad, CA, USA), followed by purification with a QIAprep Miniprep Kit (Qiagen, Germany). The DNA concentration of the plasmid standard solution was measured by fluorescence using a QuantiTPicoGreen dsDNA assay kit (Invitrogen, Carlsbad, CA, USA), as described by the manufacturer, and was related to the known molecular weight of a single plasmid molecule to calculate the number of copies. The standard curve was generated in triplicate by adjusting to the number of ITS-1 copies *μ*L^−1^. DNA (10^7^ ITS-1 copies *μ*L^−1^) was diluted in 10-fold steps. The PCR amplification efficiency (AE) was calculated from the slopes of the regressions using the equation AE = [10^(−1/slope)^] − 1 [34]. The standard curve produced for the qPCR assay revealed an amplification efficiency of 92.72% and a slope of the linear equation of −3.52, with a linear correlation coefficient of 99.85%.

### 2.6. Digital PCR (dPCR)

Digital PCR amplification reactions were performed using QuantStudio™ 3D Digital PCR (Applied Biosystems, Waltham, MA, USA), with QuantStudio Digital PCR 20 K chips, in a total volume of 16 *μ*L. The final reaction mixture contained 1x QuantStudio® 3D Digital PCR Master Mix (Life Technologies, Carlsbad, CA, USA), 0.3 *μ*M of each primer (Alum96F and Alum183R), 0.2 *μ*M of the probe Alum124T [18, 33], 4 *μ*L of DNA sample, and bdW to 16 *μ*L. The thermal cycling conditions for the amplifications were an initial denaturation step at 95°C for 10 min, followed by 40 cycles of 2 min at 60°C and 30 s at 98°C, and a final step of 72°C for 2 min. Template DNA extracted was used at 1 : 5 (standard curve), 1 : 10 (protocol 1; 500 mL of reclaimed water and protocol 2; 10 L of reclaimed water), or 1 : 20 dilutions (protocol 2; 10 L of reclaimed water). Dilutions were taken into account to calculate the number of copies per *μ*L^−1^. The amplification results, namely, the fluorescence of each partition of chip, were analyzed with QuantStudio 3D Digital PCR System Cloud Software (Life Technologies, Carlsbad, CA, USA).

### 2.7. The Modified Bailenger Method

Samples of 10 L of reclaimed water were seeded with 10 (1 sample), 50 (1 sample), and 1,000 (4 samples)* A. lumbricoides* eggs and analyzed following the modified method of Bailenger [15] by three different laboratories accredited by the three ENAC accredited laboratories (Spanish National Accreditation Body).

### 2.8. Statistics

The cycle threshold (Ct) and number of ITS-1 copies of* A. lumbricoides*  *μ*L^−1^ per number of eggs were compared using Student's *t*-test if the data sets were normally distributed (Shapiro-Wilk test; *P* > 0.05) and homoscedastic (Levene test; *P* > 0.05), or with the nonparametric Mann–Whitney test, if the data sets were not normally distributed (Shapiro-Wilk test; *P* ≤ 0.05) or did not meet the requirement of homoscedasticity (Levene test; *P* ≤ 0.05) (SPSS Inc., Chicago, IL); Student's *t*-test and Mann–Whitney test with *P* values < 0.05 were considered significant.

## 3. Results

### 3.1. qPCR

**Figure 1:**
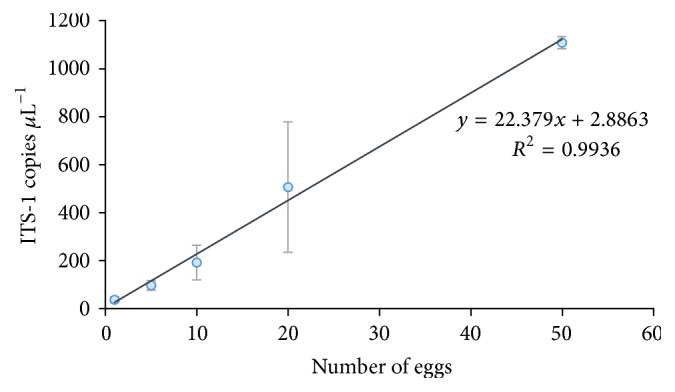
Linear regression between copies ITS-1 of* A. lumbricoides* per microliter of bidistilled water and numbers of* A. lumbricoides* eggs measured by qPCR. Each value was obtained from five replicates.

Figure 1 shows a linear regression between the ITS-1 copy numbers of* A. lumbricoides* per *μ*L^−1^ bdW (ITS-1 copies *μ*L^−1^) against different amounts of eggs in 50 *μ*L of bdW. The number of copies *μ*L^−1^ increased with the number of eggs. The negative control samples did not show amplification and the IPC and positive control amplified in all cases.

The two extraction methods with water volumes of 500 mL and 10 L, protocols 1 and 2, respectively, presented promising results (Table 1). Protocol  1 was able to detect the DNA equivalent to 1, 5, and 10* A. lumbricoides* eggs while protocol 2 was able to detect a minimum 20* A. lumbricoides* eggs, but not less than 10 eggs or 10 eggs for 2 of the batches assayed (Table 1). Quantification of* A. lumbricoides* ITS-1 copies indicated that these increased with the number of eggs with both extraction protocols. The negative control samples did not show any amplification and the positive control amplified in all cases. However, the IPC amplified with a delay of two cycles in samples following protocol  2 (10 L) compared to the one from bdW or protocol  1 (500 mL), indicating that some inhibition may have been occurring in the extracts from method 2.

Comparing both extraction methods of reclaimed water and bdW, the number of ITS-1 copies *μ*L^−1^ for eggs amount showed significant differences. Statistically significant differences between ITS-1 copy number per *μ*L from 1, 5, and 10 eggs in 50 *μ*L of bdW and 500 mL of reclaimed water were found (*P* values < 0.05). Otherwise for 10, 20 eggs in bdW compared to the 10 L extraction method showed statistically significant differences in the number of copies of ITS-1 with *P values *< 0.05 and *P* < 0.001 for 50 eggs. In both cases, higher ITS-1 copy number per *μ*L was observed in 50 *μ*L of bdW. Furthermore, statistically significant differences were also found in 10 eggs seeding 500 mL and 10 L of reclaimed water, showing *P* values of <0.05 for Ct values (data not shown) but there were no statistically significant differences for ITS-1 copies *μ*L^−1^ (*P* > 0.05).

### 3.2. dPCR

**Figure 2:**
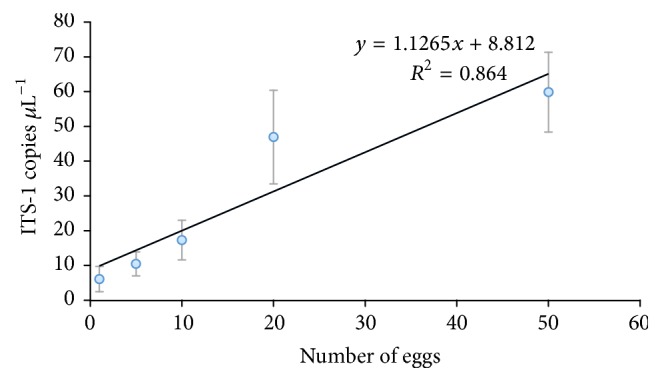
Linear regression between copies ITS-1 of* A. lumbricoides* per microliter of bidistilled water and numbers of* A. lumbricoides* eggs measured by dPCR. Each value was obtained from five replicates.

Figure 2 shows a linear regression between number of ITS-1 copies of* A. lumbricoides* per *μ*L^−1^ bdW against 1, 5, 10, 20, and 50 eggs. ITS-1 copies *μ*L^−1^ increased as the number of eggs increased with an *R*^2^ of 86.4%. Negative control did not show amplification and the IPC and positive controls amplified in all cases.

The number of ITS-1 copies *μ*L^−1^ of* A. lumbricoides* following protocol 1 showed that dPCR can detect as few as 5 eggs in reclaimed water and that these increased as the number of eggs increased (Table 2). However, following protocol  2 (10 L) dPCR did not detect any eggs in seeded reclaimed water samples (Table 2). Negative control samples did not show amplification and the IPC and positive controls amplified in all cases.

No statistically significant differences were observed for ITS-1 copies *μ*L^−1^ between eggs in bdW (1, 5, and 20) and eggs in 500 mL of reclaimed water (*P* values; 0.130, 0.086, and 0.060, resp.).

### 3.3. The Modified Bailenger Method

Table 3 shows results from the three ENAC accredited laboratories. Results indicated that the Bailenger method did not detect eggs in reclaimed water seeded with 10 and 50 eggs. When 1,000 eggs were seeded in the reclaimed water, one laboratory detected the presence of eggs but the quantified amount was very low compared with the seeded (3% of eggs).

## 4. Discussion

The use of quantitative PCR and digital PCR could be an alternative to detect* A. lumbricoides* eggs in reclaimed water. Quantitative PCR could detect DNA concentrations as low as one* A. lumbricoides *egg equivalent in 500 mL of reclaimed water (protocol 1) and 20* A. lumbricoides* eggs in 10 L of reclaimed water (protocol 2). While digital PCR detected DNA concentrations as low as five* A. lumbricoides *egg equivalents by first extraction protocol, no amplification could be detected by our second protocol. In bdW, both techniques were able to detect from one to 50 eggs with good correlation between ITS-1 copies *μ*L^−1^ bdW to number of eggs. The detection limit of the standard curve was 10 ITS-1 copies *μ*L^−1^, which is equivalent to less than one egg [35]. Similar detection limits have been reported by other authors [18].

Digital PCR was described in the 1990s and it has been focused primarily on oncology [36–38], prenatal diagnostics [39], and viruses [40–42]. Applications of dPCR for environmental samples are arising due to a high sensitivity and accurate quantification of target genes in environmental samples [43] and when there are few copies of the DNA target [36, 42, 44–46]. Some authors have reported that dPCR performed better than qPCR for DNA recovered from soils because dPCR seems to be more tolerant of PCR inhibitors [30, 38, 47].

Our study showed a good correlation with the number of eggs and dPCR in bdW (see Figure 1), but some differences were found in reclaimed water. The results showed less sensitivity of dPCR than qPCR for the DNA extractions realized by protocol 1 for 500 mL and no amplification for any of the 10 L reclaimed water samples processed via protocol 2. This could be due to increase in the reclaimed water extraction volume; then, higher inhibitory substances were extracted. Another reason might be that the volume of the template in each dPCR reaction (4/16 *μ*L) mixture is lower than in qPCR (5/25 *μ*L) and cannot be increased, which could have implied lower sensitivity [42].

One of the key points for the success of these DNA techniques is the use of an appropriate method of DNA extraction from samples of reclaimed water, as in this case, to obtain DNA of amplifiable quality avoiding possible inhibitions and low recovery of DNA that can reduce the efficiency of the PCR [16]. No amplification was observed following protocol 1 for 10 L of reclaimed water by both qPCR and dPCR (there is no data to show), probably due the coextraction of a high amount of inhibitory substances as organic matter that are still on reclaimed water after the depuration process, which could inhibit PCR and decrease its efficiency [16, 48–53]. Later, 500 mL of reclaimed water seeded with different amounts of* A. lumbricoides* eggs was extracted by the proposed protocol 1 and once optimized was adapted to DNA extraction from 10 L of reclaimed water (protocol  2). The adaptation consisted of replacing the membrane filter (higher diameter and light mesh) and the extraction buffer (CTAB + PVPP), increasing the number of filters (two filter membranes for each 10 L sample (5 L each)) and three extractions for each membrane filter. The incorporation in the DNA extraction buffer of cationic detergent CTAB [16, 54] plus polyvinylpolypyrrolidone was previously tested in DNA extractions from feces, plant, and soils to remove PCR inhibitors [18, 20, 55].

Despite the modifications, ten* A. lumbricoides* eggs were the minimum amount to be detected by using the optimized DNA extraction protocol (protocol 2). These results could indicate that the 10 L DNA extracts still showed some inhibition even after making the previously described changes in the DNA extraction protocols, as shown by the IPC reduction signal [16, 49, 56] because of the complex environmental matrices [30, 57] and the high volume of filtered water to detect helminth eggs following WHO recommendations [10]. Another important point could be the adherence of helminth eggs to different surfaces; the limited literature available on this indicates that the physical-chemical forces determining egg adherence are complex, and the use of plastic tubes and pipettes could be more effective than glass ones to reduce the adherence [58]. This could explain the loss of sensitivity in the detection of eggs in treated wastewater with respect to the bdW, due to the glass funnel used. Finally, the division of samples into two filters to obtain six aliquots per sample would further reduce the number of copies for detection.

WHO guidelines specify a threshold of egg number per specified volume for acceptable quality. For agricultural irrigation it recommends a value of ≤1 egg/L [11, 12], and recent epidemiological research work shows that a limit ≤ 0.1 egg/L is needed if children under 15 years are exposed [59]. Our results indicate that quantitation of ITS-1 copy number is imprecise, and sufficient accuracy has not been achieved such that the signal could be directly applied to estimating number of eggs based on ITS-1 estimates. But the most important finding stems from qualitative detection of ITS-1 from low egg number treatments, indicating sensitivity at a level approaching practical significance.

Respect to the detection of eggs by the modified Bailenger method [15], the results presented clearly show that the qPCR method developed was more efficient than the traditional method. Not many samples were sent; however, those results give us an idea of the Bailenger method sensitivity compared to the qPCR and dPCR. All samples were sent to ENAC accredited laboratories in order to be more objective in the conclusions obtained. As is well known, this traditional method implies many steps using sedimentation, desorption, centrifugation, and flotation of the material, after optical microscopy detection, where technical skills are needed to distinguish the helminth eggs [15]; therefore, it is not difficult to lose biological material in any step, leading to wrong results as was demonstrated with the samples that were sent. Finally, only one of three laboratories was able to detect nematode eggs in 10 L of wastewater in two samples (7 and 28 when 1,000 eggs were seeded).

Molecular techniques show promise for establishing the viability of helminths ova [35, 60, 61], several genetic targets can be studied at the same time [18], and it could achieve adequate sensitivity defined in formal guidelines. Therefore, these molecular techniques could be proposed a priori as alternatives to the traditional ones to assure the reuse of water with the appropriate health guarantees.

## 5. Conclusions

Both dPCR and qPCR can be used to detect DNA from* A. lumbricoides* eggs in reclaimed water. Quantitative PCR can detect DNA from one* A. lumbricoides* egg in 500 mL and from 10* A. lumbricoides* eggs in 10 L of reclaimed water, while dPCR can detect it from one* A. lumbricoides* egg in 500 mL of reclaimed water. The improvement of the standardization and validation of DNA extraction protocols is a very important first step in the implementation of molecular techniques in the detection of helminth eggs and in managing the required volume of water in accordance with current legislation (10 L). The main point of this paper is that qPCR has potential application for monitoring quality of treated water. Further experiments are required to demonstrate that the method can be extensively applied in multiple settings requiring nematode eggs testing. These findings support both the continued development of this technology, and the need for further work to explore its value for routine use. A second step is to be able to detect viable eggs to enable totally safe reuse of reclaimed water.

## Figures and Tables

**Table 1:** Detection of *A. lumbricoides* by qPCR from reclaimed water seeded with different amounts of *A. lumbriocoides* eggs.

Protocol 1 (for 500 mL)			Protocol 2 (for 10 L)			
Number of eggs	Mean copies *μ*L^−1^	Result	Number of eggs	Mean ITS-1 copies *μ*L^−1^	Positive extractions	Result
1	3.41 ± 0.16	Presence	10	—	—/6	Absence
1	2.54 ± 0.32	Presence	10	46.57 ± 11.03	2/6	Presence
1	4.68 ± 3.80	Presence	10	—	—/6	Absence
1	11.6 ± 2.02	Presence	10	45.16 ± 11.40	2/6	Presence
1	8.41 ± 1.93	Presence	10	48.68 ± 4.76	1/6	Presence

Average ± SD	6.14 ± 3.80			46.80 ± 1.78		

5	42.92 ± 11.27	Presence	20	61.58 ± 9.10	4/6	Presence
5	59.31 ± 13.35	Presence	20	37.96 ± 9.02	2/6	Presence
5	54.18 ± 12.94	Presence	20	42.83 ± 3.69	2/6	Presence
5	51.51 ± 4.04	Presence	20	47.67 ± 14.40	1/6	Presence
5	64.99 ± 12.8	Presence	20	47.77 ± 4.10	3/6	Presence

Average ± SD	54.58 ± 8.31			47.56 ± 8.82		

10	89.59 ± 14.4	Presence	50	123.64 ± 14.60	6/6	Presence
10	107.86 ± 4.93	Presence	50	51.76 ± 2.07	2/6	Presence
10	53.83 ± 11.04	Presence	50	39.91 ± 5.20	1/6	Presence
10	102.31 ± 11.62	Presence	50	37.64 ± 11.57	3/6	Presence
10	62.53 ± 6.84	Presence	50	56.57 ± 18.36	5/6	Presence

Average ± SD	83.22 ± 24.00			61.91 ± 35.41		

The averages are for three aliquots in 500 mL and for three aliquots of each of six different extractions per sample in 10 L. *Positive extractions*: number of positive extractions in each batch. Only from the “positive” aliquots were included in the calculation. *Presence*: Ct < 37. *Absence*: Ct > 37. Protocol  2, below 10-egg amplification was not observed.

**Table 2:** Detection of *A. lumbricoides *by dPCR from reclaimed water seeded with different amounts of *A. lumbriocoides* eggs.

Protocol 1 (for 500 mL)			Protocol 2 (for 10 L)		
Number of eggs	Mean ITS-1 copies *μ*L^−1^	Result	Number of eggs	Mean ITS-1 copies *μ*L^−1^	Result
1	4.35	Presence	10	—	NA
1	—	Absence	10	—	NA
1	—	Absence	10	—	NA
1	1.12	Presence	10	—	NA
1	7.14	Presence	10	—	NA

Average ± SD	4.2 ± 3.01				

5	13.25	Presence	20	—	NA
5	27.22	Presence	20	—	NA
5	23.77	Presence	20	—	NA
5	41.37	Presence	20	—	NA
5	7.05	Presence	20	—	NA

Average ± SD	22.53 ± 13.27				

10	16.36	Presence	50	—	NA
10	50.00	Presence	50	—	NA
10	23.30	Presence	50	—	NA
10	39.37	Presence	50	—	NA
10	28.74	Presence	50	—	NA

Average ± SD	31.55 ± 13.31				

*Presence*: more than one copy *μ*L^−1^. *Absence*: not detected; *NA*: no amplification.

**Table 3:** Number of eggs provided by different laboratories using ENAC accredited method (modified Bailenger) from *A. lumbricoides* eggs seeded in 10 L of reclaimed water.

Code number	Number of eggs seeded in 10 L of reclaimed water	Provided result by the lab
Sample 1	10	<1 egg
Sample 2	50	<1 egg
Sample 3	1,000	<1 egg
Sample 4	1,000	<1 egg
Sample 5	1,000	28 eggs
Sample 6	1,000	7 eggs
